# Productivity and Chemical–Bromatological Composition of Mombaça Grass Fertigated With Cattle Slaughterhouse Effluent With and Without Disinfection With Peracetic Acid

**DOI:** 10.1002/wer.70140

**Published:** 2025-07-17

**Authors:** Warlyton Silva Martins, Keila Cardoso Teixeira, Elizangela Alves de Freitas, Alex Sander Rodrigues Cangussu, Anna Karla dos Santos Pereira, Grasiele Soares Cavallini

**Affiliations:** ^1^ Graduate Program in Plant Production Federal University of Tocantins Gurupi TO Brazil; ^2^ Graduate Program in Chemistry Federal University of Tocantins Gurupi TO Brazil; ^3^ Graduate Program in Forestry and Environmental Sciences Federal University of Tocantins Gurupi TO Brazil; ^4^ PPGBIONORTE Federal University of Tocantins Gurupi TO Brazil

**Keywords:** fertigation, nutrition, *Panicum maximum*, sustainable, wastewater

## Abstract

Wastewater from cattle slaughterhouses is a source of nutrients for the fertigation of crops because of the presence of nitrogen and phosphorus. This study aimed to analyze the yield and nutritional composition of Mombaça grass ( *Panicum maximum* ) exposed to different doses of cattle slaughter effluent, with and without the addition of the disinfectant peracetic acid (PAA). The experiment was conducted using a completely randomized design, with eight treatments: E_100_ = 100% effluent; E_50:50_ = 50% effluent + 50% water; ET_50:50_ = 50% effluent treated with PAA + 50% water; E_75:25_ = 75% effluent + 25% water; ET_75:25_ = 75% effluent treated with PAA + 25% water; E_25:75_ = 25% effluent + 75% water; ET_25:75_ = 25% effluent treated with PAA + 75% water; and Control = 100% water, with five replicates each. Chemical and bromatological analyses of Mombaça grass showed that nitrogen, phosphorus, magnesium, and calcium levels increased progressively with an increase in the amount of effluent compared with that in plants irrigated only with water (control group). An increase of crude protein, mineral matter, and total digestible nutrients was observed, along with a reduction in the concentrations of crude fiber and ether extract in the E_100_ group. Fertigation with bovine effluent contributed to an improvement in the quality of Mombaça grass, mainly due to the increased protein amount and adequate nutrient levels. The application of PAA in the effluent contributed to the reduction of pathogens, but presented fewer benefits for the plant when compared with the effluent without PAA.

## Summary

- Increased levels of N, P, Mg, and Ca were observed in Mombaça grass with higher doses of effluent.
- Mombaça grass treated with effluent showed higher levels of digestible nutrients.
- Effluent treated with PAA maintains its nutritional benefits for use in fertigation;
- The study highlights the potential of slaughterhouse effluent for reuse of industrial wastewater.

## Graphical abstract

The study evaluated the nutritional composition of Mombaça grass exposed to different doses of cattle slaughterhouse effluent. The levels of nitrogen, phosphorus, magnesium, and calcium increased progressively with increasing effluent dose. There was a reduction in the concentrations of crude fiber and ether extract in the group fertigated with 100% effluent.

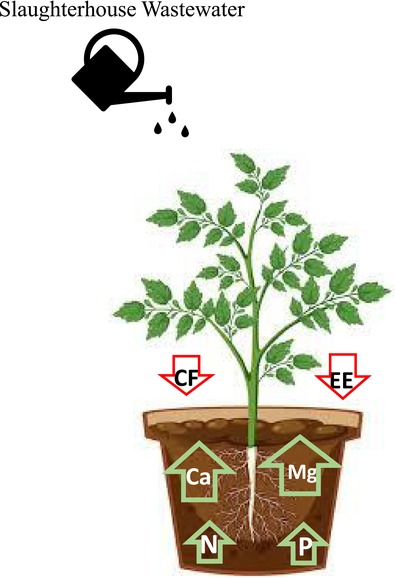

## 1 Introduction

In the search for sustainable production in agricultural systems, fertigation has emerged as a promising technique for integrating irrigation with the application of nutrients through water (Sérvulo et al. 2024). In this context, using effluents from cattle slaughterhouses as a nutrient source for fertigation has gained attention for its potential to reduce dependence on chemical fertilizers and promote the recycling of organic waste (Śniatała et al. 2024).

Matheyarasu et al. (2016) evaluated the development of plants fertigated with abattoir wastewater (AWW). Due to the abundance of nutrients (N, P, K, Ca, Mg, Zn, Fe, Al, Bo) in the soil from this wastewater, the authors observed a significant increase in biomass production and plant height in all crops evaluated and fertigated with AWW, when compared with irrigation with tap water alone.

In pastures, fertigation with these effluents represents an innovative approach that benefits both waste management and plant nutrition (Alves et al. 2023). Effluents from cattle slaughterhouses are rich in essential macro‐ and micronutrients required for plant growth, such as nitrogen, phosphorus, and potassium. However, the presence of pathogens and potentially toxic compounds also raises concerns (Menegassi et al. 2020).

Some contaminants need to be considered as reference elements when defining the application rate (Chojnacka et al. 2023), underscoring the need for disinfectants in effluent treatment. Peracetic acid (PAA) has proven to be an effective disinfectant for treating effluents intended for agricultural use because of its ability to eliminate a wide variety of pathogens with reduced environmental impacts (Pereira et al. 2025). Studies have demonstrated that, when used correctly, PAA not only considerably reduces the microbial load, including that of bacteria, viruses, and protozoa, but also decomposes into harmless byproducts such as acetic acid, water, and oxygen, thus reducing soil and water contamination risks (Souza et al. 2024).

No matter how advanced an effluent treatment technology is, it will inevitably fail if it does not consider the risks of environmental contamination (Silva et al. 2016). Therefore, the use of effluents in the fertigation of agricultural crops requires appropriate technology and care practices to minimize the contamination risks to soil and agricultural products and prevent the direct exposure of farmers to these effluents (Dhayal and Lal 2023). Therefore, detailed analyses of these effluents are essential to ensure that their application does not compromise the health of soil, plants, or animals that will consume the pasture.

However, significant knowledge gap remains regarding the long‐term effects of this practice from both agronomic and environmental perspectives. Long‐term information on nutrient accumulation, soil fertility, toxicity, and microbiological imbalances, as well as the effects on crop productivity and quality and the risk of surface water contamination, are essential for implementing alternative and sustainable practices that alleviate pressure on water resources and enable the mitigation of these impacts. Pereira et al. (2025) evaluated the effects of the application of cattle slaughterhouse effluent in the soil for periods of 5, 10, and 15 years. As a result, they obtained a supply of macro and micronutrients for pasture production and an increase in soil pH. However, the change in pH affected the reproduction of the springtail *Folsomia candida* (small arthropods that are bioindicators of soil quality).

It is recommended to determine the appropriate dosage of organic residues, based on the crop's nutrient needs to avoid adding nutrients in quantities exceeding the crop's requirement or the soil's retention capacity. Such studies are necessary to develop clear and practical guidelines that farmers can safely adopt, improve the quality and composition of pastures, reduce costs in the agronomic field and act as an alternative to chemical fertilizers. Thus, considering the potential of reusing effluents for fertigation in agriculture, in the present study, we sought to analyze the yield and nutritional composition of Mombaça grass ( *Panicum maximum* ) exposed to different doses of cattle slaughter effluent, with and without the addition of the disinfectant PAA.

## 2 Materials and Methods

### 2.1 Study Location

The experiment was conducted in a greenhouse at the Instituto Tocantinense Presidente Antonio Carlos (ITPAC) in Porto Nacional, Tocantins, Brazil, located at 10°69′57″ S, 48°38′48″ W, with an altitude of 212 m. The soil used in this experiment was classified as a Red‐Yellow Latosol, and its physical and chemical characteristics are presented in Table 1.

**TABLE 1:** Chemical characteristics of the soil in the 0‐ to 20‐cm layer in Red‐Yellow Latosol. Porto Nacional—TO.

Parameters								
pH	P (mg dm^−3^)	K (mg dm^−3^)	Ca (cmolc dm^−3^)	Mg (cmolc dm^−3^)	Al (cmolc dm^−3^)	H + Al (cmolc dm^−3^)	V (%)	O. M. (g dm^−3^)
4.2	45.3	0.05	0.03	0.22	0.21	3.05	9.16	13.5

*Note:* Clay (HMFS + Na g/kg) = 421.6; silt g/kg estimated = 91.2; total sand (sieve g/kg) = 487.2; pH in H_2_O, KCl, CaCl_2_ ratio 1:2.5; P, Na, K, Fe, Zn, Mn, Cu—Mehlich extractor 1.81; Ca, Mg, Al—KCl extractor 1 mol L^−1^; H + Al—calcium acetate extractor 0.5 mol/L, pH 4.20; V (%) = base saturation; O.M. = organic matter.

The amount of limestone required to correct the soil pH was calculated using the base saturation method, resulting in a recommended application of 3.19 t ha^−1^. During the experimental period, no topdressing was applied to the grass.

### 2.2 Slaughterhouse Wastewater

Bovine effluent was collected from a municipal slaughterhouse in Gurupi, Tocantins, Brazil, where an average of 1200 head of cattle are processed monthly. The effluent was collected directly from the treatment plant after undergoing primary and secondary treatment (untreated with PAA). In the laboratory, it was further treated with commercial PAA (15%) at a concentration of 10 mg L^−1^. Subsequently, both the untreated and PAA‐treated effluents were examined for turbidity, solids (dissolved, suspended, and total), conductivity, chemical oxygen demand, dissolved oxygen, total coliforms, *Escherichia coli* concentrations, pH, color, temperature, and total nitrogen (Baird et al. 2017) parameters. Elemental analysis was performed to evaluate the chemical characteristics of the samples. The physicochemical and microbiological characteristics of the bovine effluent used in the experiment are presented in Table 2.

**TABLE 2:** Physicochemical and microbiological characterization of the bovine effluent used in the experiment.

Parameters	Untreated effluent	Effluent treated with PAA (10 mg L^−1^)
pH	7.21	7.11
Temperature	26.8	26.06
COD (mg L^−1^)	480	370
DO (mg L^−1^)	3.55	5.0
Conductivity (μS cm^−1^)	914.5	843.3
Salinity (mg L^−1^)	435	400
Turbidity (NTU)	60.6	60.6
True color (uC)	386	216
Total nitrogen (mg L^−1^)	1147	972
Sodium (mg L^−1^)	141.11	136.47
Potassium (mg L^−1^)	31.3	30.9
Magnesium (mg L^−1^)	9.86	9.92
Calcium (mg L^−1^)	62.11	59.86
Molybdenum (mg L^−1^)	0.01	0.02
Phosphorus (mg L^−1^)	15.54	15.71
Zinc (mg L^−1^)	0.16	0.21
Iron (mg L^−1^)	0.32	0.38
Copper (mg L^−1^)	0.01	0.01
Nickel (mg L^−1^)	0.01	0.02
Manganese (mg L^−1^)	0.23	0.23
*E. coli* (CFU/100 mL)	1350	< 1
Total Coliforms (CFU/100 mL)	8000	100
PAA residual (mg L^−1^)	—	0.1

Abbreviations: COD = chemical oxygen demand; DO = dissolved oxygen.

### 2.3 Preparation of Mombaça Grass Samples

#### 2.3.1 Planting

Mombaça grass was as the selected plant for this study because of its high nutrient requirements, preference for deep soils with good drainage, and adaptation to the local climate, characterized by high temperatures and over 1000 mm of annual rainfall. Mombaça grass was planted in 10‐L pots, using seeds with a germination percentage of 25.5%, applied to the soil using a sieve. Five grams of seeds were used, corresponding to 25 g per experimental plot.

The experiment was conducted using a completely randomized design, with eight treatments (E_100_ = 100% effluent; E_50:50_ = 50% effluent + 50% water; ET_50:50_ = 50% effluent treated with PAA + 50% water; E_75:25_ = 75% effluent + 25% water; ET_75:25_ = 75% effluent treated with PAA + 25% water; E_25:75_ 25% effluent + 75% water; ET_25:75_ = 25% effluent treated with PAA + 75% water; Control = 100% water) and five replicates.

The crop water requirements in all pots were calculated using daily reference evapotranspiration (ET0), determined using the Penman–Monteith method as ET0 × Kc = CET (Allen et al. 2006), based on data obtained through internet research. Determining crop evapotranspiration (CET) is necessary for efficient water use. CET was calculated using daily values of the crop coefficient (Kc). The accumulated water depths were applied daily to replace the CET. A nitrogen concentration of 40 kg ha^−1^ was used as a reference for calculating water depths. Water depth and fertigation were determined by weighing the pots, with the weight difference used to calculate the volume required to restore field capacity.

#### 2.3.2 Fertigation

The application of the effluent (with and without PAA treatment) began when the grass reached approximately 15 cm in height. For effluent application, a hydraulic system was used to store and distribute wastewater to the experimental plots. Irrigation was carried out every 2 days, meaning that effluent was applied to the plots once every 2 days. This continued until the required amount was reached for each plot. During the irrigation process, two harvests were made in Mombaça grass at intervals varying between 25 and 35 days, depending on the plant growth, to evaluate productivity.

#### 2.3.3 Assessment of Chemical–Bromatological Properties

The grass samples were dried in an oven with forced air circulation at 65°C for 72 h. The leaves were then ground in a Willey‐type mill, and the resulting powder was stored in labeled and sealed polyethylene bags. Subsequently, the samples were subjected to chemical–bromatological and foliar analyses to determine the levels of crude protein (CP), crude fiber (CF), ether extract (EE), mineral matter (MM), total digestible nutrients (TDN), N, P, K, Ca, Mg, Cu, Zn, Fe, Mn, and Na in the dry matter.

The Kjeldahl method was used to determine the total N content. The amounts of P, K, and Na were measured following nitric–perchloric acid digestion of the samples, using spectrophotometry and flame photometry. The concentrations of the other nutrients were evaluated using plasma emission spectrophotometry, following the guidelines of the Brazilian Agricultural Research Corporation, Embrapa (da Silva et al. 2017). The percentages of CF, CP, MM, and EE were determined according to the protocols described by Caputi (2017). The data were subjected to an analysis of variance. Means were compared using Dunnett's and Tukey's tests at a 5% probability. Statistical analyses were performed using the SISVAR program (Ferreira 2011).

## 3 Results and Discussion

**FIGURE 1:**
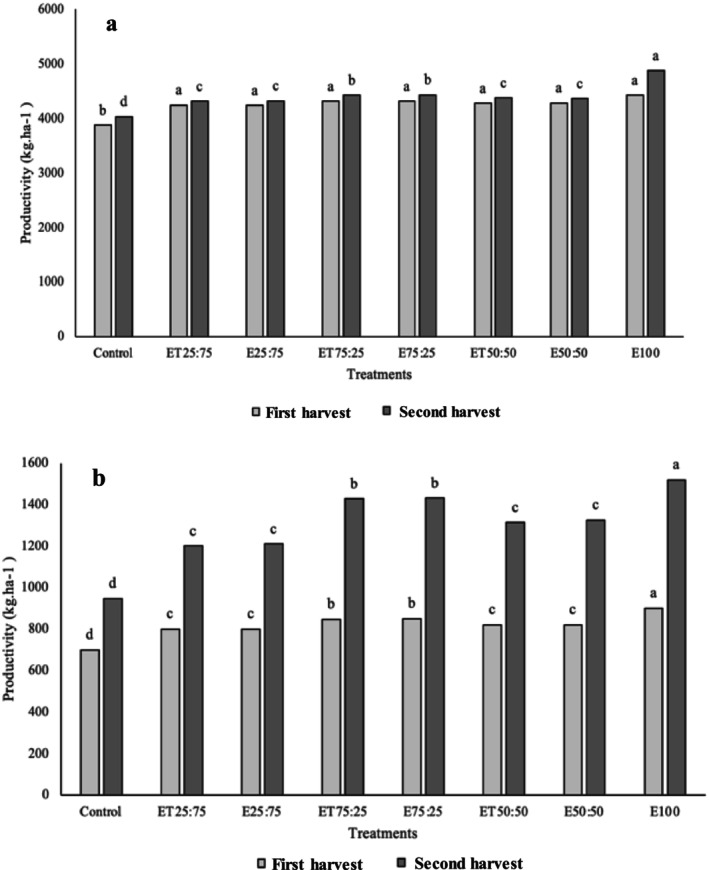
(a) Productivity of green and (b) dry matter of Mombaça grass leaves in different harvests as a function of fertigation with bovine slaughterhouse effluent with and without disinfection with PAA (E_100_ = 100% effluent; E_50:50_ = 50% effluent + 50% water; ET_50:50_ = 50% effluent treated with PAA + 50% water; E_75:25_ = 75% effluent + 25% water; ET_75:25_ = 75% effluent treated with PAA + 25% water; E_25:75_ 25% effluent + 75% water; ET_25:75_ = 25% effluent treated with PAA + 75% water; water = control). Values followed by the same letter do not differ statistically by the Tukey's test, at 5% probability.

The evaluation of the productivity of Mombaça grass subjected to fertigation treatments with bovine slaughterhouse effluent, with and without disinfection using PAA, showed significant variations between the treatments (Figure 1).

The results presented in Figure 1a reveal a significant increase of 550 kg ha^−1^ in green matter productivity with the application of fertigation using the effluent during the second harvests of Mombaça grass. Regarding dry matter, productivity was low in all treatments during the first harvests, with the plants treated only with water (control) showing the lowest productivity, reaching an average of 947.4 kg ha^−1^ of dry matter. This value is below the 1200 kg ha^−1^ previously reported by Müller et al. (2002). For the second harvest, plants subjected to lower effluent doses exhibited a better visual appearance, although no nutritional deficiencies were observed in the control plants. Additionally, these plants showed higher productivity in both green matter (Figure 1a) and dry matter (Figure 1b) compared with that in the first harvests. The best results were obtained by the plants treated with ET_75:25_, E_75:25_, and E_100_, which received higher doses of bovine effluent and, consequently, a greater amount of nutrients.

The results indicate that, for this type of soil, grass, and the tested levels, the effluent from the cattle slaughterhouse was effective in significantly increasing productivity (Figure 1). This increase is associated with the nutrients in the effluent that became available in the soil. However, the use of the effluent treated with PAA resulted in a decrease in available N levels, in addition to influencing the solubility and availability of other nutrients, such as P and K, by altering the pH of the effluent (Table 2).

In a study on the use of cattle slaughterhouse effluents, da Silva Neto et al. (2010) reported an increase in the fresh and dry mass production of *Brachiaria brizantha* “Marandu” with increasing effluent doses. Similarly, Serafim and Galbiatti (2012) analyzed the production and chemical composition of *B. brizantha* “Marandu” fertilized with pig effluent and observed a significant increase in fresh and dry mass production with increasing effluent doses.

Lozano et al. (2015) conducted a study on the reuse of poultry slaughterhouse effluent to irrigate Mombaça grass and reported a significant increase in both fresh and dry matter production with increasing effluent doses. In contrast, dos Santos et al. (2013), while conducting a similar study using dairy effluents to irrigate Mombaça grass on sandy soil, concluded that dairy effluents exhibit the potential to provide the nutrients required for forage crops.

Drumond et al. (2006) reported that applying swine effluent to Tifton 85 grass significantly increased the amount of dry matter produced as the amount of effluent applied increased. At a dose of 200 m^3^ ha^−1^, the amount of dry matter produced was twice as high as that in the control treatment using only water.

Similarly, da Silva et al. (2018) found that dry matter in Vaquero grass significantly increased after the application of 450 m^3^ ha^−1^ year^−1^ of rendering effluent compared with that in the control treatment without fertilization, reaching 23 t ha^−1^ of dry matter. These results indicate that effluents have the potential to replace mineral fertilizers in the production of dry matter from forage plants.

Regarding the nutrient concentrations in the leaves of Mombaça grass, significant differences were observed among treatments for N, P, K, Ca, and Mg (Table 3).

**TABLE 3:** Nutrient concentrations in Mombaça grass leaves as a function of fertigation with cattle slaughterhouse effluent with and without PAA disinfection.

Treatment	Concentration									
	N (%)	P (%)	K (%)	Ca (%)	Mg (%)	S (%)	Cu (mg/kg)	Fe (mg/kg)	Mn (mg/kg)	Zn (mg/kg)
Water	0.80	0.08	0.96	0.36	0.02	0.02	5	395	80	66
ET_25:75_	0.80	0.90*	1.04*	0.40	0.05	0.02	5.5	405	83	65
E_25:75_	0.80	0.90*	1.14*	0.44	0.05	0.02	5.3	405	83	65
ET_50:50_	0.80	0.80*	1.00*	0.42	0.06	0.03	5.5	405	85	67
E_50:50_	0.80	0.90*	1.04*	0.44	0.06	0.03	5.5	405	85	67
ET_75:25_	1.27*	0.70*	1.00*	1.00*	0.05	0.02	5.5	405	85	67
E_75:25_	1.32*	0.92*	1.00*	1.04*	0.08*	0.02	5.5	405	85	67
E_100_	1.40*	0.24*	1.52*	1.20*	0.10*	0.03	5.5	405	85	67

*Note:* E_100_ = 100% effluent; E_50:50_ = 50% effluent + 50% water; ET_50:50_ = 50% effluent treated with PAA + 50% water; E_75:25_ = 75% effluent + 25% water; ET_75:25_ = 75% effluent treated with PAA + 25% water; E_25:75_ 25% effluent + 75% water; ET_25:75_ = 25% effluent treated with PAA + 75% water; water = control. In each harvest in the columns, the means with an asterisk differ from the control treatment by Dunnett's test, at 5% probability.

The P content in the grass leaves, at different concentrations of bovine effluent (Table 3), varied between 0.24% and 0.92%, showing that the use of effluent in the irrigation significantly increased the P content in the aerial parts of the plant compared with those in the control (water). According to Freitas et al. (2007), these values are sufficient to meet the nutrient needs of beef cattle, dairy cattle, and sheep. Malavolta et al. (1997) reported that these values are considered adequate, as P levels in the leaves must range between 0.16 and 1.1 dag kg^−1^.

The reduction in P content in the grass leaves with the use of 100% untreated cattle effluent in irrigation (E_100_) may have been caused by mineral nutrients present in the effluent, particularly N, which, when present in cationic form, hinders the absorption of K. Similarly, when present in anionic form (NO_3_^−^), it can hinder the absorption of P, as the cation–anion balance affects the absorption of nutrients. Thus, relatively high quantities of N may be present in the effluent in anionic form, hindering the absorption of other anions (Uçgun and Altindal 2021).

Moreover, a reduction in grass P content was also observed in the ET_50:50_ and ET_75:25_ treatments, wherein the effluent treated with PAA was used. These variations can be attributed to the use of PAA, which acts as a powerful oxidant during the disinfection process and promotes reactions that can alter the chemical form of P present in the effluent (Collivignarelli et al. 2017). Specifically, PAA can oxidize organic P compounds, converting them into relatively less soluble forms or precipitating them as insoluble phosphates, thereby reducing their bioavailability in soil. However, the combination of chemical fertilization with fertigation using cattle effluent could be a viable alternative to adjust P levels in plants, resulting in a reduction in the costs of mineral fertilizers.

There was a large variation in K levels in plant leaves, even within the same treatment (Table 3). According to Malavolta et al. (1997), K levels in Mombaça grass ranged from 1.43% to 1.84%. It was observed that the K concentration was within this range only in the E_100_ treatment. None of the other treatments could achieve satisfactory K levels in the plants, despite showing statistically significant differences compared with those in the control treatment.

Average N levels increased as the applied effluent dose exceeded 50%, demonstrating the relatively high presence of this nutrient when the effluent was more concentrated. The N levels in the leaves were higher in the ET_75:25_ (1.27%), E_75:25_ (1.32%), and E_100_ (1.4%) treatments (Table 3) than the values reported by Malavolta et al. (1997), which ranged from 1.13 to 1.5%. Freitas et al. (2007) reported that the application of different doses of N (70, 140, 210, and 280 kg ha^−1^) applied as urea to Mombaça grass in the Cerrado of Goiás, resulted in grass N levels between 1.38 and 1.68 dag kg^−1^. Thus, our results agree with the findings of Freitas et al. (2007), indicating a rapid release of N from the bovine effluent used in this study. Furthermore, we observed no adverse effects of other ions present in bovine effluent on N absorption.

Significant N levels were observed only in the treatments with higher effluent volume. The PAA present in the ET_75:25_ treatment (1.27% N), due to its oxidizing properties, can promote the oxidation of N compounds (Tong et al. 2025; da Silva et al. 2020), such as ammonia (NH_3_) and nitrite (NO_2_^−^), into nitrates (NO_3_^−^), which can be readily leached (Ding et al. 2021). The decrease in N content can compromise the agronomic value of the effluent, especially in agricultural systems with a predominance of forage plants, which depend on these residues as important sources of N fertilization (Delevatti et al. 2019). Additionally, PAA treatment altered the pH balance of the effluent, further influencing the form and solubility of N.

Ca levels in the grass treated with effluent were higher than those in the control group only at the highest effluent concentrations (ET_75:25_, E_75:25_, and E_100_). According to Malavolta et al. (1997), Ca and Mg levels in the leaves are considered adequate when they range between 0.4% and 1.02%, and 0.12% and 0.22%, respectively. In our study, however, Mg levels invariably remained below the values described by Malavolta et al. (1997) in all treatments. Acidification of the effluent by PAA can change the soil pH after application, further influencing the solubility and mobility of Ca^2+^ and Mg^2+^, which may reduce their uptake by plants (Jing et al. 2024).

Dhayal and Lal (2023) recommended avoiding fertilizers rich in K to prevent interference with the absorption of Ca and Mg by plants, which could result in lower yields. Because the E_100_ treatment had a higher concentration of K than other elements, it is recommended to use it as a reference in real‐world management situations to prevent potential nutritional problems. Although PAA is relatively less reactive with K compared with other nutrients, such as Ca and Mg, it may indirectly impact the availability of K^+^ ions in the soil after the application of the treated effluent.

Furthermore, Sérvulo et al. (2024) reported that the K present in organic fertilizers is in ionic form, not as stable organic compounds, making the nutrients readily available for absorption by plants. Therefore, the K present in the effluent will be immediately available in the soil after application, which may lead to adverse effects. The interaction of K with other nutrients present in the soil, such as Ca and Mg, could be affected by the changes in chemical conditions following the application of the effluent. An adequate balance between these cations is essential to avoid antagonism, which may impair K absorption by plants.

The fertigation treatments combining effluent (treated or untreated) with water demonstrated intermediate results. Even when the effluent was properly diluted, satisfactory results were achieved in terms of the adequate nutritional quality of Mombaça grass. Table 4 presents the statistical differences in the average bromatological characteristics of Mombaça grass subjected to fertigation with bovine effluent.

**TABLE 4:** Averages of the bromatological characteristics of Mombaça grass: CP—crude protein; CF—crude fiber; EE—ethereal extract; MM—mineral matter; TDN—total digestible nutrients for the different fertigation treatments with bovine slaughterhouse effluent with and without disinfection with peracetic acid.

Treatments	Crude protein (%)	Crude fiber (%)	Ether extract (%)	Mineral matter (%)	TDN (%)
Water	6.30 d	30.5 a	2.20 a	5.60 c	52.60 c
ET_25:75_	8.20 b	29.50 b	1.80 b	6.60 b	61.00 b
E_25:75_	8.10 c	29.50 b	1.80 b	6.60 b	61.00 b
ET_75:25_	8.27 b	29.50 b	1.90 b	7.40 a	60.80 b
E_75:25_	8.13 c	29.50 b	1.80 b	7.40 a	60.30 b
ET_50:50_	8.40 b	29.50 b	1.90 b	6.80 b	60.20 b
E_50:50_	8.30 b	29.50 b	1.80 b	6.80 b	60.10 b
E_100_	9.70 a	28.30 c	1.40 c	7.50 a	77.30 a

*Note:* (A) Green matter productivity. (B) Dry matter productivity (E_100_ = 100% effluent; E_50:50_ = 50% effluent + 50% water; ET_50:50_ = 50% effluent treated with PAA + 50% water; E_75:25_ = 75% effluent + 25% water; ET_75:25_ = 75% effluent treated with PAA + 25% water; E_25:75_ = 25% effluent + 75% water; ET_25:75_ = 25% effluent treated with PAA + 75% water; water = control). TDN: total digestible nutrients. Means followed by different letters in the vertical row differ from each other at 5% by Tukey's test.

The CP levels showed a significant effect for the different treatments. The E_100_, 100% effluent treatment (without PAA), yielded significantly higher CP levels (approximately 9.7%) than those obtained in the other treatments. The lowest CP levels were found in the control treatment, with a statistically significant increase observed in grass protein content after the administration of the effluent to the grass. The results are consistent with those reported by Benett et al. (2008), who studied the effects of different doses and sources of N source on the productive and qualitative characteristics of Marandu grass and concluded that CP levels above 9% can occasionally be observed with an increase in N doses. Thus, it can be concluded that CP content in a plant depends essentially on the amount of N absorbed, a nutrient present in high concentrations in the bovine effluent used in the present study.

Moreover, CP levels below 7% limit animal production, resulting in a lower voluntary consumption, reduced digestibility, and a negative N balance (Machado et al. 1998). Thus, it can be suggested that Mombaça grass would satisfactorily meet the minimum N requirements for ruminants in any of the treatments applied (Table 4), as the increase in N levels in the tissues of effluent‐fertigated plants will markedly improve their nutritional quality when the total N is converted into CP content. High CP levels are desirable to meet the protein requirements of ruminants, as pasture is the most economical way to meet the protein demand of animals (Silva et al. 2018).

It should be noted that there are few studies on the bromatological composition of forage plants subjected to fertigation with bovine effluent. In the present study, the E_100_ treatment resulted in the best values for CF, ether extract (EE), and total digestible nutrients (TDN) at 28.3%, 1.4%, and 77.3%, respectively, compared with the other treatments. Moreover, the E_100_, E_75:25_, and ET_75:25_ treatments resulted in higher averages of mineral matter, ranging from 7.4% to 7.5%, than those in the other treatments.

Regarding CF content, a reduction of 28.3% was observed in the CF content in plants subjected to the E_100_ treatment compared with that of 30.5% in the CF content in plants irrigated with water (control). Fiber is a term used to describe an aspect completely related to nutrition and is characterized as the non‐digestible or slowly digestible portion of the food that fills the gastrointestinal tract; a higher CF content normally indicates greater lignification of the fiber. In this case, the effluent treatments proved relatively more effective in improving the nutritional quality of feed, as they reduced the CF content in feed. A higher CF content corresponds to a lower energy level in the feed, as CF is considered indigestible.

In a study evaluating the nutritional quality of Marandu grass fertilized with treated sewage effluent, Santos et al. (2017) observed that the continuous use of this effluent, together with increasing doses, resulted in forage with better nutritional quality, with CP levels of approximately 16% and neutral detergent fiber (NDF) averaging 60%. Conversely, Andrade et al. (2012) found an average NDF of 76.7% for Vaquero grass fertilized with 600 kg ha^−1^ of N per year, a value higher than that obtained in the present study.

The EE content in feed represent the most readily digestible carbohydrates, such as sugars, starch, and pectin. Thus, the higher EE content in the control treatment must be related to the pectin content in the cell wall, as CF was also higher in this treatment. According to Van Soest (1994), pectin is an amorphous polysaccharide present in the cell wall, which is completely and rapidly degraded by ruminal microorganisms. The results showed that Mombaça grass had excellent nutritional value, marked by high CP levels, especially when cultivated in bovine effluent. Thus, the effluent positively influenced the nutritional value of Mombaça grass, providing higher CP content and lower forage CF.

The TDN content in feed represents the quantity of nutrients available for absorption. Plants treated with 100% bovine effluent without PAA (E_100_) exhibited relatively high TDN value, implying that fertigation using high effluent levels can help improve nutrient availability in the resultant feed.

## 4 Conclusions

Mombaça grass treated with cattle effluent, both with and without PAA disinfection, showed significant changes in the amounts of green and dry matter produced during the first and second harvest. The presence of N, P, Mg, and Ca increased progressively with an increase in the amount of effluent used, whether treated with PAA or untreated, compared with that in plants of control group irrigated only with water. Variations in the nutrient content, particularly N and P, were observed when comparing effluents treated with PAA to untreated effluent as the oxidation power of PAA interfered with the bioavailability of these nutrients.

An increase in the amounts of CP, mineral matter, and TDN was observed, along with a reduction in CF and EE concentrations in the E_100_ group treated with 100% effluent in the absence of PAA. The adoption of fertigation with bovine effluent improved the quality of Mombaça grass, primarily by increasing protein levels and ensuring the availability of adequate nutrient levels.

Regarding the application of PAA, a nutritional decrease can be observed when compared with the same effluent dosages without PAA, however, the benefits related to application safety, both for human health and environmental preservation, justify the disinfection stage because it presented superior results to the control.

## Author Contributions

**Warlyton Silva Martins:** investigation, Writing – original draft. **Keila Cardoso Teixeira:** investigation. **Elizangela Alves de Freitas:** Investigation. **Alex Sander Rodrigues Cangussu:** conceptualization, visualization, writing – review and editing. **Anna Karla dos Santos Pereira:** conceptualization, visualization, writing – review and editing. **Grasiele Soares Cavallini:** conceptualization, visualization, methodology, supervision.

## Ethics Statement

The authors have nothing to report.

## Consent

The authors have nothing to report.

## Conflicts of Interest

The authors declare no conflicts of interest.

## Data Availability

The data that support the findings of this study are available from the corresponding author upon reasonable request.

## References

[wer70140-bib-0001] Allen, R. G. , L. S. Pereira , D. Raes , and J. Smith . 2006. Evapotranspiración del Cultivo: Guias para la Determinación de los Requerimientos de Água de los Cultivos. FAO.

[wer70140-bib-0002] Alves, D. K. M. , M. B. Teixeira , F. N. Cunha , F. R. Cabral Filho , G. N. Cunha , and C. L. L. de Andrade . 2023. “Grain Yield of Maize Crops Under Nitrogen Fertigation Using Wastewater From Swine and Fish Farming.” Agronomy 13, no. 7: 1834. 10.3390/agronomy13071834.

[wer70140-bib-0003] Andrade, A. S. , L. C. D. Drumond , M. F. Appelt , D. D. Moreira , F. C. de Araújo , and P. I. V. G. God . 2012. “Crescimento e Composição Bromatológica de Tifton 85 e Vaquero em Pastagens Fertirrigadas.” Glass Science and Technology 5: 56–68.

[wer70140-bib-0004] Baird, R. B. , A. D. Eaton , and E. W. Rice . 2017. Standard Methods for the Examination of Water and Wastewater. 23rd ed. American Water Works Association.

[wer70140-bib-0005] Benett, C. G. S. , S. Buzetti , K. S. Silva , A. F. Bergamaschine , and J. A. Fabricio . 2008. “Produtividade e Composição Bromatológica do Capim‐Marandu a Fontes e Doses de Nitrogênio.” Ciência e Agrotecnologia 32, no. 5: 1629–1636. 10.1590/S1413-70542008000500041.

[wer70140-bib-0037] Caputi, B. , (coord.) 2017. “Compêndio Brasileiro de Alimentação Animal.” In Matérias‐primas e ingredientes. edited by P. São , 5th ed., 204. SINDIRAÇÕES; ANFAL; CBNA.

[wer70140-bib-0006] Chojnacka, K. , D. Skrzypczak , D. Szopa , G. Izydorczyk , K. Moustakas , and A. Witek‐Krowiak . 2023. “Management of Biological Sewage Sludge: Fertilizer Nitrogen Recovery as the Solution to Fertilizer Crisis.” Journal of Environmental Management 326: 116602. 10.1016/j.jenvman.2022.116602. 36375429

[wer70140-bib-0007] Collivignarelli, M. , A. Abbà , G. Alloisio , E. Gozio , and I. Benigna . 2017. “Disinfection in Wastewater Treatment Plants: Evaluation of Effectiveness and Acute Toxicity Effects.” Sustainability 9, no. 10: 1704. 10.3390/su9101704.

[wer70140-bib-0008] da Silva, A. E. , A. Fontana , A. d. S. Melo , et al. 2017. In Manual de Métodos de Análise de Solo, edited by P. C. Teixeira , G. K. Donagemma , A. Fontana , and W. G. Teixeira , 3rd ed. Embrapa.

[wer70140-bib-0033] da Silva, W. P. , T. D. Carlos , G. S. Cavallini , and D. H. Pereira . 2020. “Peracetic Acid: Structural Elucidation for Applications in Wastewater Treatment.” Water Research 168: 115143. 10.1016/j.watres.2019.115143. 31590037

[wer70140-bib-0009] da Silva Neto, S. P. , J. E. C. da Silva , A. C. dos Santos , J. G. D. Castro , V. P. Dim , and A. D. S. Araújo . 2010. “Características Agronômicas e Nutricionais do Capim‐Marandu em Função da Aplicação de Resíduo Líquido de Frigorífico.” Acta Scientiarum. Animal Sciences 32, no. 1: 9–18. 10.4025/actascianimsci.v32i1.6247.

[wer70140-bib-0010] da Silva, P. L. , D. G. de Oliveira , M. C. Melo , D. D. Camargo , and L. C. D. Drumond . 2018. “Avaliação de Parâmetros Produtivos do Capim Vaquero Fertirrigado com Água Residuária de Agroindústria.” Journal of Engineering and Exact Sciences 4, no. 3: 0353–0358. 10.18540/jcecvl4iss3pp0353-0358.

[wer70140-bib-0035] Delevatti, L. M. , A. S. Cardoso , R. P. Barbero , et al. 2019. “Effect of Nitrogen Application Rate on Yield, Forage Quality, and Animal Performance in a Tropical Pasture.” Scientific Reports 9, no. 1. 10.1038/s41598-019-44138-x. PMC6527677 31110320

[wer70140-bib-0011] Dhayal, D. , and K. Lal . 2023. “Nutritional Status of Wastewater Irrigated Soil Under Lemongrass (*Cymbopogon flexuosus*).” Pharmaceutical Innovation 12, no. 2: 211–217. 10.22271/tpi.2023.v12.i2c.19148.

[wer70140-bib-0034] Ding, Y. , X. Huang , Y. Li , et al. 2021. “Nitrate Leaching Losses Mitigated With Intercropping of Deep‐Rooted and Shallow‐Rooted Plants.” Journal of Soils and Sediments 21, no. 1: 364–375. 10.1007/s11368-020-02733-w.

[wer70140-bib-0012] dos Santos, P. M. , A. C. dos Santos , and J. E. C. da Silva . 2013. “Resíduo de Laticínio em Pastagem de Capim Mombaça: Atributos Químicos da Forragem e do Solo.” Semina: Ciências Agrárias 34, no. 1: 377–390. 10.5433/1679-0359.2013v34n1p377.

[wer70140-bib-0013] Drumond, L. C. D. , J. R. Zanini , A. d. P. A. Aguiar , G. P. Rodrigues , and A. L. T. Fernandes . 2006. “Produção de Matéria Seca em Pastagem de Tifton 85 Irrigada, com Diferentes Doses de Dejeto Líquido de Suíno Ask ChatGPT.” Engenharia Agrícola 26, no. 2: 426–433. 10.1590/S0100-69162006000200010.

[wer70140-bib-0014] Ferreira, D. F. 2011. “Sisvar: A Computer Statistical Analysis System.” Ciência e Agrotecnologia 35, no. 6: 1039–1042. 10.1590/S1413-70542011000600001.

[wer70140-bib-0015] Freitas, K. R. , B. Rosa , A. J. Ruggiero , et al. 2007. “Avaliação da Composição Químico–Bromatológica do Capim Mombaça (*Panicum maximum* Jacq.) Submetido a Diferentes Doses de Nitrogênio.” Bioscience Journal 23: 1–10.

[wer70140-bib-0036] Jing, T. , J. Li , Y. He , et al. 2024. “Role of Calcium Nutrition in Plant Physiology: Advances in Research and Insights Into Acidic Soil Conditions ‐ A Comprehensive Review.” Plant Physiology and Biochemistry 210: 108602. 10.1016/j.plaphy.2024.108602. 38608506

[wer70140-bib-0016] Lozano, C. S. , T. U. Tonello , E. C. Bortoletto , M. A. Araújo , and A. P. Tonello . 2015. “Resposta do Capim Mombaça (*Panicum maximum* cv. Mombaça) Submetido à Aplicação de Água Residuária de Abatedouro de Aves.” Enciclopédia Biosfera 11, no. 22: 3796–3805. 10.18677/Enciclopedia_Biosfera_2015_267.

[wer70140-bib-0017] Machado, A. O. , U. Cecato , R. T. Mira , L. A. F. Pereira , and J. C. Damasceno . 1998. “Evaluations of Chemical Composition and In Vitro Dry Matter Digestibility of Cultivars and Accesses of *Panicum maximum* Jacq. Under Two Cutting Heights.” Revista Brasileira de Zootecnia 27: 1057–1063.

[wer70140-bib-0018] Malavolta, E. , Vitti, G. C. , & Oliveira, S. A. 1997. Avaliação do Estado Nutricional das Plantas: Princípios e Aplicações. 2nd ed. (A. B. para P. da P. e do Fosfato, Ed.).

[wer70140-bib-0019] Matheyarasu, R. , N. S. Bolan , and R. Naidu . 2016. “Abattoir Wastewater Irrigation Increases the Availability of Nutrients and Influences on Plant Growth and Development.” Water, Air, & Soil Pollution 227, no. 8: 253. 10.1007/s11270-016-2947-3. 27440946 PMC4932140

[wer70140-bib-0020] Menegassi, L. C. , F. Rossi , L. D. Dominical , et al. 2020. “Reuse in the Agro‐Industrial: Irrigation With Treated Slaughterhouse Effluent in Grass.” Journal of Cleaner Production 251: 119698. 10.1016/j.jclepro.2019.119698.

[wer70140-bib-0021] Müller, M. d. S. , A. L. Fancelli , D. Dourado‐Neto , A. García y García , and R. F. López Ovejero . 2002. “Produtividade do *Panicum maximum* cv. Mombaça Irrigado, Sob Pastejo Rotacionado.” Scientia Agricola 59, no. 3: 427–433. 10.1590/S0103-90162002000300003.

[wer70140-bib-0022] Pereira, M. A. B. , A. K. d. S. Pereira , T. D. Carlos , et al. 2025. “Ecotoxicity and Chemical Characterization of Tropical Soil Under Different Periods of Exposure to Cattle Slaughterhouse Effluent.” Environmental Science: Advances 4, no. 5: 763–770. 10.1039/D4VA00373J.

[wer70140-bib-0023] Santos, G. O. , R. T. de Faria , G. A. Rodriguês , G. D. F. Dantas , A. B. Dalri , and L. F. Palaretti . 2017. “Forage Yield and Quality of Marandugrass Fertigated With Treated Sewage Wastewater and Mineral Fertilizer.” Acta Scientiarum Agronomy 39, no. 4: 515. 10.4025/actasciagron.v39i4.32828.

[wer70140-bib-0024] Serafim, R. S. , and J. A. Galbiatti . 2012. “Effect of the Application of Swine Wastewater in *Brachiaria brizantha* cv Marandu.” Revista Colombiana de Ciencias Animales 4: 185–203.

[wer70140-bib-0025] Sérvulo, A. C. O. , D. Sandri , and M. L. G. Ramos . 2024. “Reusing Livestock Farming Wastewater for Tifton 85 Irrigation: Productivity, Morphological, and Bromatological Indicators.” Ambiente e Agua—An Interdisciplinary Journal of Applied Science 19: 1–14. 10.4136/ambi-agua.2954.

[wer70140-bib-0026] Silva, J. G. D. , J. M. R. da Luz , J. Henrique , J. J. de Carvalho , and J. E. C. da Silva . 2018. “Domestic Wastewater for Forage Cultivation in Cerrado Soil.” Journal of Agricultural Science 10, no. 10: 248. 10.5539/jas.v10n10p248.

[wer70140-bib-0027] Silva, J. G. D. , J. J. de Carvalho , J. M. R. da Luz , and J. E. C. da Silva . 2016. “Fertigation With Domestic Wastewater: Uses and Implications.” African Journal of Biotechnology 15, no. 20: 806–815. 10.5897/AJB2015.15115.

[wer70140-bib-0028] Śniatała, B. , H. E. Al‐Hazmi , D. Sobotka , J. Zhai , and J. Mąkinia . 2024. “Advancing Sustainable Wastewater Management: A Comprehensive Review of Nutrient Recovery Products and Their Applications.” Science of the Total Environment 937: 173446. 10.1016/j.scitotenv.2024.173446. 38788940

[wer70140-bib-0031] Souza, A. K. N. , J. Paggiaro , W. S. Martins , A. K. S. Pereira , D. H. Pereira , and G. S. Cavallini . 2024. “Theoretical‐Experimental Evaluation of the Effects of Fe3+ Ions in the Disinfection of Water Supply by Peracetic Acid.” Discover Water 4, no. 1: 70. 10.1007/s43832-024-00126-5.

[wer70140-bib-0032] Tong, Y. , Wang, X. , Zhang, Y. , J. Xu , and C. Sun . 2025. “Reactive Species in Peracetic Acid‐based AOPs: A Critical Review of Their Formation Mechanisms, Identification Methods and Oxidation Performances.” Water Research 272: 122917. 10.1016/j.watres.2024.122917. 39671863

[wer70140-bib-0029] Uçgun, K. , and M. Altindal . 2021. “Effects of Increasing Doses of Nitrogen, Phosphorus, and Potassium on the Uptake of Other Nutrients in Sweet Cherry Trees.” Communications in Soil Science and Plant Analysis 52, no. 11: 1248–1255. 10.1080/00103624.2021.1879122.

[wer70140-bib-0030] Van Soest, P. J. 1994. Nutritional Ecology of the Ruminant. 2nd ed. Cornell University Press.

